# Supplementary material for: Learning curve and surgical outcome of robotic assisted colorectal surgery with ERAS program
Source: Sci Rep. 2022 Nov 29;12:20566. doi: 10.1038/s41598-022-24665-w (PMC9709162; doi:10.1038/s41598-022-24665-w)
Supplement: Supplementary file 1 — Supplementary Information 1. [file 41598_2022_24665_MOESM1_ESM.zip › LSC Raw data-Ñ[▒K/078/H_ñw╝╨░O▒Kñσ.pdf]

正式摘要(電子簽章：000000190187150)

W52 / W52 ☐英文標題(注意:無英文姓名-請補病人基本檔!) ☐【讀取】憑證

臺中榮民總醫院

出院病歷摘要

轉入號： 轉出號：

[redacted]

**(9)**入院日期	109/04/22 (2020) 直腸外科 W52 - 091
**(10)**轉科日期	
**(11)**出院日期	109/05/06 (2020) 住院天數計 14 日

診斷

- 入院：1.Ca of upper rectum
  2.Hypertension
  3.Hydrocephalus s/p VP shunt in CSMUH 2 years ago.
- 出院：1. Upper rectum adenocarcinoma s/p laparoscopic anterior resection on 2020/4/24, pT3N0
  2. Hypertension
  3. Hydrocephalus s/p left LP shunt in CSMUH in 2018
  # Removed from abdomen on 2020/4/24, placed back into abdomen on 2020/05/04

主訴

- bloody stool for months

病史

- This 82-year-old female has history of
  1.Ca of upper rectum
  2.Hypertension
  3.Hydrocephalus s/p VP shunt in CSMUH 2 years ago.
  According to his son, she suffered from bloody stool for months. Then she was brought to DaLi Jen-Ai Hospital where colonoscopy revealed rectum ca. Then she went to our OPD for second opinion. Surgical intervention was suggeted. After discussed with patient, she was admitted for scheduled operation.

體檢發現

- Anal inspection:
  Recto digital examination:
  Sigmoidoscopy:
  Anoscopy:
  Abdomen:
  - General appearance :
    - easy looking , conscious clear , without cardiopulmonary distress
    - Vital signs自動帶入date 體溫(BT) 脈搏(P) 呼吸(R) 血壓(BP) 身高(BH) 體重(BW) BMI
      20200422 36.1℃ 84次/分 18次/分 154/75mmHg 154.1cm 57.7kg 24.3
    - Vital signs: B.P. 154/75 mmHg , P.R. 84 /min , R.R. 18 /min , B.T. 36.1 ℃
  - HEENT :
    - Eyes: no anemic conjunctiva, no icteric sclera, normal EOM
    - ENT: no otorrhea, no rhinorrhea, no erythematous mucosa, no injected tonsil, no deformity
  - Neck: supple, freely movable, trachea midline,no deformity, no engorged jugular vein , no palpable thyroid or lymph node enlargement
  - Chest:
    - Inspection: Grossly normal, symmetric and fully expansion, no accessory muscle used, no operation scar, no spider angiomata, no gynecomastia
    - Palpation: no palpable mass or nodule, no subcutaneous emphysema, no tenderness

-
    - Ausculation: clear breathing sound
- Heart:
    - Percussion: normal heart size
    - Ausculation: regular heart beat, normal S1, S2, no gallop, no murmur
- Abdomen:
    - Inspection: in
        - flat contour, symetrically, no scar, no stria, no dilated veins, no rashes
    - Ausculation: normoactive bowel sound, no abdominal bruits
    - palpation: soft, no muscle guarding or rigidity, no local tenderness, no rebounding pain, no palpable mass, no palpable pulsation
    - Percussion: no shifting dulllness, no hepatomegaly, no splenomagaly
-

Peripheral pulsation :	CA	BA	RA	FA	PA	PTA	DPA
Right side :	++	++	++	++	++	++	++/+++
Left side :	++	++	++	++	++	++	++/+++

- Genito-anus: no tenderness or palpable mass lesion or rectal shelf, normal external genitalia, normal anal tone, brown stool

CRS CONDITION

- CEA: 4.49 ng/ml
-
    - Lung

手術日期及方法

- 手術日期及方法20200424 1. Laparoscopic LAR with end-to-end anastomosis, double stapling, endo GIA 60/4.1mm, CDH 29mm. Re-inforcement of silk: nil, air tight test : done
  2. Bilateral donuts: intact
  3. Re-peritonealization : No
  4. Takedown of splenic flexure: No
  5. Proximal margin: 15cm, distal margin: 2.5cm
  6. Vessel ligation: IMA above left colic artery
  7. 19 Fr. hemovac drain x 1 at the pelvis
  20200424 Pull-out abdominal catheter
  20200504 Revision of CSF shunt

住院治療經過

- After admission, patient received operation as planned. Her LP shunt was temporarily removed out the abdomen to avoid possible intra-abdominal infection.
  Her appetite gradually improved and the LP shunt tube was placed back into abdomen on 5/04.
  Under relative stable condition, we would discharge her and arrange OPD for F/U.

合併症

一般檢查

- 累積報告 :

DATE	TIME	ALB	TP	BIL.T	BIL.D	ALKP	AST	ALT	LDH	CHOL	TG	HDL-C	LDL	UA	NH3	AMY
1090422	1628	4.0														
1090427	0959			0.32	0.08		15	5								
1090430	0916	3.1		0.28				11								
1090504	0951	3.0		0.3			12	9								

DATE	TIME	WBC	RBC	HGB	HCT	MCV	PLT	BLST	NEUT	BAND	SEG	LYM	MON	EOS	BAS
1090423	0907			10.0	29.9										
1090427	0855	8040	3.82	10.3	31.0	81.2	262		72.6			14.2	7.5	5.2	0.5
1090430	0912	6450	3.56	9.6			282		69.0			14.4	8.2	7.8	0.6
1090504	0929	7120		9.1	28.5		358		69.4			15.0	7.4	7.4	0.8

DATE	TIME	NA	K	CL	CA	BUN	CREAT	Mg	P	CRP	ESR	PCT	LIPASE	LACTATE
1090422	1628	141	4.3	107	8.7	22	1.81							
1090427	0959	137	4.3		8.1	14	1.35							
1090430	0916	140	4.6		8.0	25	1.46							
1090504	0951	143	4.0		7.9	23	1.38							

DATE	TIME	GLUCOSE	PAB	TRF	ZN	CU	KETONE	CK	CKMB	PT-P	PT-C	APTT-P	APTT-C	TRO-T
1090504	0916									10.5	10.3	29.3		

DATE	TIME	SP-GR	PH	PROT	GLUC	KETON	BILI	UROB	OB	NITR	LEU	RBC	WBC	EPCEL
1090423	2218	1.011	7.0	Negative	Negative	Negative	Negative	0.2	1+	Positive	1+	5-10	5-10	0-1

特殊檢查

病理報告

- 活體組織切片病理報告　手術日期：**109/04/24**　病理號：**S0911452**

診斷醫師:楊啟順 報告日期:109/04/29
Pathologic diagnosis:
1. Rectum, laparoscopic low anterior resection --- Adenocarcinoma, moderately differentiated. Tumor invaded to perirectal soft tissue.
The bilateral cut ends and perirectal lymph nodes (0/19) are free of tumor.
2. IMA root, dissection --- Unremarkable lymph nodes (0/2) and fibrovasculoadipose tissue.
3. Rectum, cut end, distal, resection --- Unremarkable lymph node (0/1) and colon mucosa.
Ancillary study for diagnosis:
IHC stains for PMS2 and MSH6 are done on section C. Both markers are preserved.
Prognostic and predictive factor:
1. Tumor size: 4x2.7 cm.
2. Depth of invasion: Pericolorectal tissue.
3. Histologic grade: G2: Moderately differentiated.
4. Angiolymphatic invasion: Not identified.
5. Perineural invasion: Not identified.
6. Tumor deposits (discontinuous extramural extension): Absent.
7. Macroscopic tumor perforation: Not identified.
8. Surgical Margins: a) Proximal Margin: Uninvolved by invasive carcinoma, distance of tumor from margin: 13.8 cm. b) Distal Margin: Uninvolved by invasive carcinoma, distance of tumor from margin: 0.7 cm. c) Radial or Mesenteric Margin: Uninvolved by invasive carcinoma, distance of tumor from margin: 3 cm at least.
9. Lymph node status: Uninvolved (0/22).
10. Extranodal involvement: Not applicable.
11. Tumor regression grading S/P CCRT: No known presurgical therapy.
12. Type of polyp in which invasive carcinoma arose: Not identified.
13. Additional pathologic findings: Not identified.
14. Pathological TNM Stage: pT3N0 (According to the eighth edition, American Joint Committee on Cancer Staging Guidelines for Tumors).
15. TNM descriptors: Not applicable.
Gross description:
The specimen consists of 1) a segment of colon, 18.5 cm in length and 3 cm in proximal cut end circumference, 5.5 cm in distal circumference, and perirectal soft tissue. A gray tan firm annular elevated and centrally ulcerative tumor, 4x2.7 cm, is noted, 0.7 cm to the distal cut end. On cross section, the tumor invades to the muscular layer with more than 3 cm to the radial margin. The both cut ends are free of tumor grossly. 2) IMA root, a piece of tan yellow soft tissue, 4.2x3.1x1.6 cm. 3) distal cut end, a piece of tan mucosa-covered soft tissue, 2.2x1.7x0.6 cm. Representative part for section: A) proximal cut end B) distal cut end C-F) tumor G) radial margin H) specimen 2 J) specimen 3 K-M) lymph nodes.
Note:
1. The interpretation of MMR panel and recommended studies are as below: No loss of nuclear expression of MMR proteins: low probability of microsatellite instability-high (MSI-H). Please note: There are exceptions to the above IHC interpretations. These results should not be considered in isolation, and clinical correlation with genetic counseling is recommended to assess the need for germline testing.
2. This case has been peer reviewed by two doctors.
#T-59600_2 #M-81403_2 2 1105 S0000F
#T-C4560_2 #M-09183_2 0 1105 000000

其它

出院時情況

- 3.改門診治療

出院指示

出院計劃

主治醫師 2062F 蔣鋒帆 / 住院醫師 2301H 林陳彥

正式摘要**(電子簽章：000000190187150)**

W52 / W52

doctorRank:B
